# Adolescents' Perception of Causes of Obesity: Unhealthy Lifestyles or Heritage?

**DOI:** 10.1016/j.jadohealth.2012.08.015

**Published:** 2012-12

**Authors:** Helen Gonçalves, David A. González, Cora P. Araújo, Ludmila Muniz, Patrícia Tavares, Maria C. Assunção, Ana M.B. Menezes, Pedro C. Hallal

**Affiliations:** a Postgraduate Program in Epidemiology, Universidade Federal de Pelotas, Pelotas, Brazil; b Postgraduate Program in Nutrition, Universidade Federal de Santa Catarina, Santa Catarina, Brazil; c Epidemiological Research Center, Universidade Federal de Pelotas, Pelotas, Brazil

**Keywords:** Adolescence, Obesity, Medicalization, Qualitative research, Fatness, Social control, Cohort studies

## Abstract

**Purpose:**

To evaluate adolescents' perception of the causes of obesity, with emphasis on differences according to nutritional status and socioeconomic position.

**Methods:**

We conducted qualitative research including 80 adolescents belonging to the 1993 Pelotas (Brazil) Birth Cohort Study, and their mothers. We classified adolescent boys and girls into four groups (girls–obese, girls–eutrophic, boys–obese, and boys-eutrophic) according to body mass index for age and sex, and systematically selected them according to family income at age 15 years. Research techniques included semistructured interviews and history of life. Topics covered in the interviews included early experiences with weight management, effect of weight on social relationships, family history, eating habits, and values.

**Results:**

Low-income obese adolescents and their mothers perceive obesity as a heritage, caused by family genes, side effects of medication use, and stressful life events. However, low-income eutrophic adolescents emphasize the role of unhealthy diets on obesity development. Among the high-income adolescents, those who are obese attribute it to genetic factors and emotional problems, whereas those who are eutrophic mention unhealthy diets and lack of physical activity as the main causes of obesity.

**Conclusions:**

Perceptions of the causes of obesity in adolescents from a middle-income setting vary by gender, socioeconomic position, and nutritional status. Whereas some blame genetics as responsible for obesity development, others blame unhealthy diets and lifestyles, and others acknowledge the roles of early life experiences and family traditions in the process of obesity development.

**Implications and Contribution**

Low-income obese adolescents and their mothers perceive obesity as a heritage/identity. Eutrophic adolescents, however, emphasize the importance individual characteristics or gene family (internal causes) on obesity development regardless of income level. Among high-income adolescents, those who are obese attribute it to genetic factors and life events (external causes).

Demographic, economic, social, and nutritional transitions that occurred in the past decades shifted public health paradigms worldwide [1]. The growing prevalence of overweight and obesity in virtually all age ranges is one of the key characteristics of these transitions [2]. In Brazil, from 2006 to 2011, the prevalence of overweight increased from 43% to 49%; the rise in the prevalence of obesity in the same period was from 11%–16% [3]. The increasing prevalence of obesity represents an enormous public health threat. It leads to an increased risk of several noncommunicable diseases, which are the leading causes of deaths worldwide [4]. Because of its high and growing prevalence, obesity has been considered an epidemic (or even pandemic) of global proportions [5–8], although some criticize the use of these labels in the obesity context [9,10]. Despite divergence opinions about the label that should be attached to obesity, campaigns against it have increased in recent years [11].

Despite the growing recognition of obesity as a public health challenge, it has been pointed out that our understanding of the multiple factors influencing a person's risk—particularly, a population's risk of developing obesity—is still insufficient [12]. A key criticism of current approaches against obesity is the excessive medicalization of obesity. This medicalization process has been questioned for favoring the dissemination of distorted ideal weight notions and discrimination against obese subjects, creating norms that go much beyond the diet-related ones [9,10]. There are many studies on how medicalizing a problem might be a reductive process [10,13–15]. Given the link between habits and behaviors of the modern industrialized world established by the medical discourse, the term “obesity” has become popularly recognized in its most individualistic aspect, which leads to discrimination and even bullying against obese subjects [14,16–18]. The focus on individual behaviors ignores the well-known influences of environment and megatrends on health, and may lead to an underestimation of the influence of macrosocietal and microsocietal components on weight gain [13,16]. All of these factors influence how the society and individual subjects perceive obesity.

Previous research emphasized the roles of gender and socioeconomic status in the process of obesity development [10,19,20]. An example of the complexity of gender–socioeconomic status effects on obesity is that fact that in Brazil, obesity is more frequent among high-income men and low-income women compared with their peers [21]. Another critical element in the obesity development equation is the adolescence phase. In many societies such as Brazil that have experienced rapid economic growth, subjects who were exposed to food deprivation in infancy and childhood entered adolescence in a completely different scenario of food abundance, particularly ultraprocessed foods that are highly associated with obesity risk [22]. Because obesity tends to track from adolescence to adulthood [23], preventing its development in this phase is essential for public health.

Qualitative studies in this field may provide insights into the perceptions that people have in relation to obesity [24–27]. In this context, the aim of this article was to evaluate how perceptions about the causes of obesity differ between (1) high- versus low-income adolescents, (2) boys versus girls, and (3) obese versus eutrophic adolescents.

## Methods

The city of Pelotas in Southern Brazil has approximately 330,000 inhabitants and is located in the extreme south of the south region of the country. The south region was the one which experienced the most dramatic rise in the prevalence of obesity over the past 10 years [28]. The 1993 Pelotas (Brazil) Birth Cohort Study included all hospital births taking place in the city during that calendar year. Newborns were measured and mothers (n = 5,249) were interviewed on a number of sociodemographic and general health variables. The cohort members were subsequently followed up on numerous occasions from infancy until age 18 years. Detailed methodological information on the study is available elsewhere [29].

For this study, we interviewed 80 adolescents from the cohort, aged 16–17 years, and their mothers using a qualitative approach. We used anthropometric information to stratify individuals by nutritional status using body mass index (BMI) for age according to the World Health Organization standards [30]. Anthropometric data were available for 4,349 adolescents. Adolescents with BMI between the fifth and 84th percentiles at 11 and 15 years of age were considered eutrophic (n = 2,527), and those with a BMI ≥95th percentile at both time points were considered obese (n = 216). Adolescents who changed nutritional status and those classified as thin or at risk of overweight were not eligible for this study (n = 1,606). We then stratified the cohort database by gender and obesity status (obese versus eutrophic adolescents). In each of the four BMI–sex groups (eutrophic girls, n = 1,334; obese girls, n = 79; eutrophic boys, n = 1,193; and obese boys, n = 137), individuals were organized in ascending order according to their family income at age 15 years. In each group, we systematically selected a sample of 20 adolescents. The interval for selection was defined as n/20, where n is the number of adolescents in each group, and we selected the starting point at random among individuals in the first interval for each group. At the end, this sampling process led to a total sample size of 80 adolescents (Table 1 ). Two of 80 respondents refused to participate. In addition, we could not locate two adolescents after three attempts on different times and days, and we therefore replaced them with adolescents in subsequent order according to the ascending family income list and BMI–sex group.

Table 2 summarizes the anthropometric and socioeconomic characteristics of the adolescents. Data are presented as mean and standard deviation or as median and interquartile range, depending on the distribution of the variables. Because of the asymmetry and heterogeneity of variances, we assessed differences using the Kruskal-Wallis test. The level of statistical significance was set at .05.

The Ethical Committee of the Federal University of Pelotas Medical School approved the study protocol. This qualitative study used research techniques including semistructured interviews, history of life with adolescents and their mothers, and informal conversations with family friends who were at home during at least one of the home visits. Two trained interviewers recorded and performed all interviews at home. Students hired for this function transcribed the recorded conversations. All names used throughout this article are pseudonyms, to ensure confidentiality.

**Figure 1:**
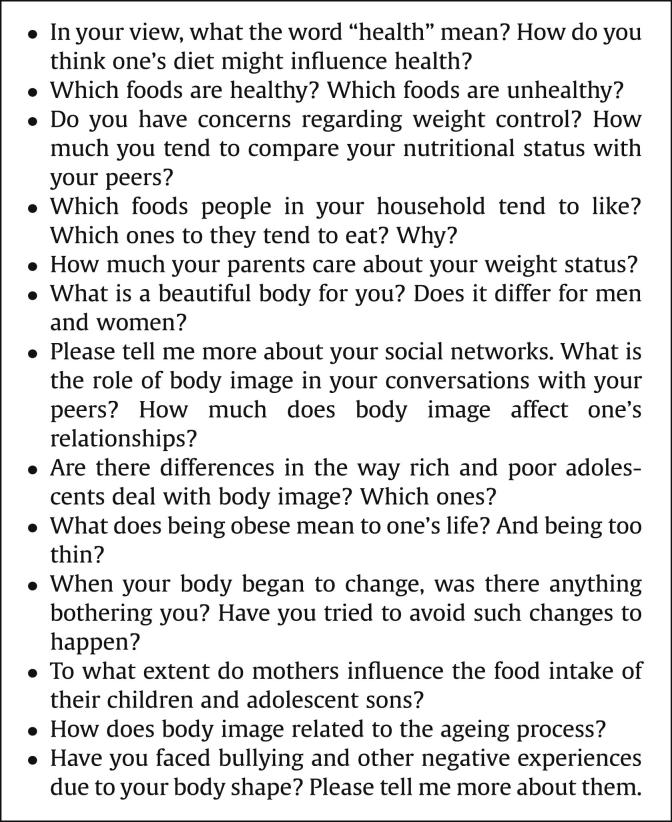
Items and questions used to guide interviews.

Fieldwork lasted 7 months (September 2009 to March 2010). The interview guide (Figure 1 ) included topics on obesity, diet, and culture, and open questions aimed at exploring the experiences of the participants. Early experiences with weight management, the impact of nutritional status on social relationships, family history of obesity, attitudes toward obesity, eating habits, values attributed to food, and body image were some of the topics discussed during the interviews.

Before starting the fieldwork, we also conducted pilot interviews with adolescents and adults not pertaining to the cohort study, to test our research techniques and improve our interview guide. We asked about topics at the most appropriate moments during the conversation, without a predefined sequence.

Before the first contact with the cohort members, the interviewers were unaware of the adolescent's nutritional status and family income. The first interview was designed to develop rapport; observe interactions; and elicit family histories, living circumstances, food practices (purchase, preparation, and eating patterns), and family health perceptions and practices. In the second visit, the interviewers conducted a semistructured interview dealing with specific, individualized issues that emerged during the first contact. On average, each interview lasted 1.5 hours. Each new theme was explored in later interviews and used to develop the interview guide for other adolescents and mothers. This means that each theme that emerged could be explored in further detail in future interviews with the same person or with another cohort member. We conducted three to four interviews with each adolescent and one or two with the mother.

The research team audio-taped, transcribed verbatim, and discussed all interviews in bimonthly meetings that took place throughout the fieldwork. Data collection was considered to be completed when interviews started providing no additional information that contributed meaningfully to the topic under investigation [31].

**Figure 2:**
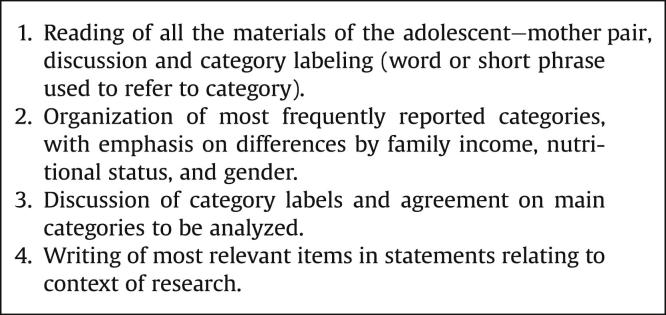
Overview of data analysis process.

The lead author and the interviewer examined all interview materials independently, to identify emerging categories and themes after the fieldwork was completed. After their independent reviews, they had meetings aimed at defining the analytical categories. Particular attention was given to (1) exploring in-depth situations in which the adolescent was dissimilar from his peers, and (2) identifying new research questions. Figure 2 describes the analytical steps. In short, the last steps in the analyses were to prioritize new readings and codify all interviews to create a list of representative categories of each subject within each analytical category. We developed, refined and grouped these categories. Although this is a lengthy process, it enabled us to use a more interpretative approach, taking into account important socioanthropological concepts such as medicalization [10,14,15], habitus [32]. and social control [33].

## Results and Discussion

One of the main findings of our interviews was that adolescents and mothers acknowledge the role of early life experiences in obesity development. Interestingly, subjects do not blame either emotional problems or biophysiological processes as single causes of obesity. In fact, adolescents and mothers easily accept that the process of obesity development has both emotional and biophysiological causes, both of which are part of the biomedical model of obesity development. Our findings also showed that low-income obese adolescents and their mothers perceive obesity to be a heritage caused by family genes, side effects of medication use, and stressful life events. Eutrophic adolescence, however, emphasize the role of unhealthy diets on obesity development. Among high-income adolescents, those who are obese attribute it to genetic factors and emotional problems, whereas those who are eutrophic mention unhealthy diets and lack of physical activity as the main causes of obesity (Table 3 ). Table 3 presents only answers that were provided by at least five of 10 adolescent–mother pairs.

Initially, we selected two groups of the main categories for in-depth analysis. The first group, entitled Family Meals and Fat: Heritage and Identity, discusses how family memories and traditions, as well as genetic factors, create a family identity that influences the food people eat. The second group, entitled Sociocultural Pressures, Unwanted Effects, and Life Events, discusses the role of peer pressure, aesthetics, and stressful events in determining what people eat and how they want to look.

### Family, meals and fat: heritage and identity

The messages given by health professionals and media about healthy eating and lifestyle relate to a series of complex concepts on food, obesity, and fat that were pointed out in respondents' statements. One convincing answer was about the importance of family habits, acquired through family meals, in determining current eating patterns. Adolescents and mothers of both income strata emphasized the importance of pleasurable eating and the social benefits of family meals. Daily meals are composed of dishes such as “Grandma's meat,” “Dad's beans,” or the “Son's french fries.” Adolescents and their mothers do not report being concerned with the health consequences of this type of traditional family eating. In fact, these traditional meals are clearly identified as positive much more than negative.

The common idea of genetics as the origin of obesity was initially upheld among high- as well as low-income obese adolescents of both sexes. Having obese parents and relatives was given as an explanation for current nutritional status. Respondents also mentioned that future generations were likely to continue experiencing the problem, because it is a “family issue.”

In the lower-income segment, the way in which food is prepared and offered is not seen as an explanation for obesity occurrence. Low-income people in our sample mentioned that food is “for filling the stomach,” whereas among high-income families, the “nibbling” concept was mentioned several times. The genetic inheritance reference—“tendency to gain weight”—disregards, in part, the responsibility of the individual and the family for the mode of preparation, type of cooking, and nutritional quality. Daniel (BMI, 35.6 kg/m^2^; low-income) has lunch and dinner with the family. He likes pasta and fried food prepared by his mother and calls himself “chubby since childhood.” Because the father is “fat”, the family believes that “the entire side of his family is like that.” Because of this, the family has never worried about the child's weight, and besides being like the father, “He does everything a thin person does and never had health problems.” The association between biology and weight o fatness strengthens the adolescent's belonging to the group (“took after my family”). In this sense, biology reaffirms an important link between the family and the subject. Another finding from this discourse is that fatness is understood as a disease only when a clinical alteration is diagnosed.

To extenuate the current standards of weight and height, low-income families reinforce the importance of being naturally what they are. They refute the way they believe high-income people eat: “nibble and waste food to maintain appearances instead of eating heartily.” Family members of this group also recognized economic achievements by food abundance. Many parents' stories involved food deprivation. Being able to eat much or at any time is also a sign of prestige, economic, and social power for adolescents. Having a healthy body is not considered the same as being thin.

In the high-income segment, heredity is considered a part of the biological process that the young obese should resist and overcome with effort and support. Genetics is seen as a complicating factor, but not as a definitive one. A male adolescent explained, “I don't blame genetics; I blame myself for not taking care of myself.” The idea that the individual should react to the environment works as an ally in the weight loss process, which is established as soon as boys feel aesthetically and affectively rejected by girls, and vice versa. All of them mentioned daily menu changes: unhealthy food being replaced with healthier options. Some mothers reported adopting the same diet.

The valorization of a thin, healthy, and muscular body in this group as indicators of education, discipline, beauty, and self-control stood out in the practice of encouraging children to exercise and consult a health professional. Wealthy obese girls were less likely to engage in exercise because of they were ashamed of their body. They were constantly dieting to look thinner, hardly went out, and had low self-esteem. Boys did not mind being vain, desiring more muscles, and having less fat. Of the 20 high-income obese respondents, 14 (nine boys and five girls) had lost enough weight to change nutritional status from 11 to 15 years of age. Among the 20 low-income obese adolescents, only one girl was no longer classified as obese in that period.

Family members of obese adolescents from the lower-income group are also theoretically supportive when their children want to lose weight. However, because they consider genetics (gene family) to be main reason for obesity, they eventually weaken the adolescent's desire to resist traditional diets. We noticed that in this group, diets rarely changed, with only a modest increase in the report of eating vegetables. For these mothers, fighting against genetics in favor of a thin body pattern will require more obstacles than those faced by children without a family history of obesity. Weight loss diets are seen as punishment more than as a positive lifestyle choice. A mother of a low-income adolescent on a weight loss diet reported that her daughter concealed eating popcorn with condensed milk. Another mother, also of low-income, understood her daughter's grief over the weight scale. After the girl lost and regained 33 kg, she tried to convince herself that she didn't mind her fatness. Despite the daughter's discontentment with her image, the mother proudly described her consumption: “[My husband] buys food every week. A lot of food comes into our home! [What kind of food?] Pizza, pastry, lasagna, those heavy things [strong], soda, cake.” These statements clearly show that speaking about lifestyle changes and obesity with this group is to overlook a process of family achievements, identity, and class habitus in favor of what is not always a prestigious standard for all.

Mothers of high-income male and female adolescents adopted different strategies to ensure the success of a diet, by providing one or more foods associated with the pleasure of the previous diet. One of them said, “You can take away other things but not Coca-Cola! You have to identify these pleasure things. You associate diet with starvation; suffering … it should not be that way!”.

### Sociocultural pressures, unwanted effects, and life events

After exhausting claims about intergenerational transmission of fat in the body, some mothers in the lower-income group also pointed out the causal relation between weight in adolescence and events in childhood. The use of natural fortifiers (herbs and plants mixture, ferrous sulphate, and phosphoric acid) and vitamins prescribed by them or by doctors are some examples. Luana had weight and height lower than other neighborhood children. Her mother, who was from a humble family, used to be questioned by other mothers about the daughter's supposed prematurity in childhood. Feeling pressed and given the comparisons with “bigger” children, she decided to give Luana a traditional tonic before meals. Ten years later, in adolescence, the daughter pointed out the consequences of the medicine: “It's because of the medicines that I took as a child!” The mother agrees: “It's not because she eats, she's fat. It's because she took a lot of vitamin fortifier when she was a little girl.”

However, adolescents and their mothers do not always agree about the etiology of obesity. Obese girls from both income groups tended to blame their mothers for excessive concerns with undernutrition in infancy and childhood, and stated that these concerns were responsible for their being obese as adolescents. Among high-income boys, however, mothers were more likely to blame themselves, stating that current unhealthy diet patterns were responsible for the fact that their adolescents were obese.

A “large” or “strong” body—which some would call fat—may actually be desirable by families. That is how most low-income obese boys saw themselves or wanted to be seen. “Chubby boys,” as they described themselves, wished to reduce the belly and shape the arm muscles. For them, the size of the belly or waist circumference was what defined fat and risk of disease. Unlike what the nine high-income, formerly obese boys said, losing weight would not be enough to gain admiration and feel good. Shaping muscles with exercise would be also needed.

Girls and boys from low-income families often reported that a beautiful body “must have flesh”; a skinny woman or man is as rejected as an obese one (because of a sagging belly or because the person does not walk “normally”). Low-income eutrophic girls reported great efforts to gain weight—“look hot.” They wanted to have a body like models or popular singers, who wear scanty clothes to project shapely bodies with aesthetic intervention. For the wealthiest income group, this slender body pattern fit into the “disgusting,” “fat,” and “ugly” categories.

Some mothers of obese adolescents from both income groups highlighted other concerns about obesity, by mentioning that their sons could face difficulties such as discrimination in the job market, discrimination in social relationships, harmful long-term health consequences of obesity, and the possibility of isolation from friends.

Another key component in the obesity equation is emotional health. Interviewees considered that weight gain may derive from anxiety, depression, or the absence of a family member. For many high-income and low-income families, the death of a close family member, and especially the divorce of parents, caused changes in eating and emotional behavior (e.g., depression). Family members from five low-income and seven high-income adolescents noticed higher food consumption after the parents' divorce, and, in two other cases (both low-income), because of a grandparent's death. This association between eating and emotional disorders [34] guaranteed the family that mourning or changes in the house dynamics could be alleviated by food. In low-income families, mothers often adopted the “let him or her eat” as a means of facilitating adaptation into a new neighborhood or low contact with the father. Among the high-income group, going to psychologists was more frequently reported as a means of adapting to changes and losses, resulting from the power of assimilation biomedical discourse.

After the two main sections defined a priori, we decided to devote a short section to the issues of medicalization, popular knowledge, and social problems, summarizing the implications of obesity biomedical discourses on the perception of the population about the problem.

### Medicalization, popular knowledge, and social problems

Over the past 3 decades there has been a decrease in malnutrition and absolute poverty rates, and fast economic growth in Brazil [35]. The latter provides greater access to and consumption of goods and services [36]. In the country, obesity has increased in all age groups [3,28], and estimates for future years are alarming [37]. Health professionals use media programs to inform the population about how to eat, live, and have a healthy body. This health promotion, primarily focused on diet and physical activity, is based on the disease epidemic or pandemic notion of obesity, and is an example of how social issues permeate body image and diets that, once medicalized, are targeted by biomedical or biopolitical conceptions of social control [10,14,15,38].

It is not surprising that obese and eutrophic adolescents from both family income groups and sex usually mentioned terms such as “metabolism,” “calories,” “carbohydrates,” and “BMI” when talking about obesity, overweight, and fat. These terms, previously used only by health professionals, are now widely employed in the media and other communication channels. Felipe, who is from a high-income family, justified eating two small chocolate bars by saying that the amount of calories in those two bars was not extreme, and therefore he would not become fatter as a result of its consumption. By emphasizing the caloric equation, adolescents were able to minimize being guilty for eating unhealthy foods. Clearly, each family and individual finds a particular strategy to deal with the abundance of information available, depending on the body–health–disease conceptions learned over time. A previous study showed that both researchers and the media are likely to blame individuals more than the environment and genetics factors for obesity development [11,39].

In some conversations, we observed an apparent contradiction: “Genetics is an excuse!” “He or she is obese because he or she eats a lot!” Disregarding the term “genetics” now ratifies the excuse and portrays more subtle social forms of pressure, regulation, and biomedical power on what is good or bad, and normal or abnormal. Body weight becomes a moral issue, emphasizing personal disabilities and responsibilities. Explicitly, eating fattens, and the lack of control causes the weight to exceed. In sequence, mentioning ”to eat a lot” was justified by a lack of willpower, which is frequently attributed as being lazy, unwilling, psychologically or emotionally weak, and slow. Among obese adolescents, these adjectives were clearly enunciated by nine adolescents who had gone on a diet and by five who had not. All of the eutrophic ones pointed to these characteristics. One of them said, “Gluttony and sloth—a near fatal combination.” The notion that obesity is a sign of weakness instead of a genetic or biological issue leads to an individualizing model in which the subject is seen as the only person responsible for his or her own health [5,10,11].

Our report shows that the perceptions about the causes of obesity in adolescents from a middle-income setting vary by gender, socioeconomic position, and nutritional status. Whereas some blame genetics as being responsible for obesity development, others blame unhealthy diets and lifestyles, and others acknowledge the roles of early life experiences and family traditions in the process of obesity development. The challenge of reducing the rising rates of obesity should not be underestimated or taken lightly. Health systems should not expect that the availability of scientific information will automatically lead to behavioral change. A multisectoral and multimethodological approach is urgently needed in the obesity arena if we are serious about tackling it.

## Figures and Tables

**Table 1:** Family income in analytical categories (N = 80)

	Girls		Boys		*p* value^a^
	Eutrophic (n = 20)	Obese (n = 20)	Eutrophic (n = 20)	Obese (n = 20)	
	(median [p25–p75])	(median [p25–p75])	(median [p25–p75])	(median [p25–p75])	
Family income at age 11 years (R$)^b^	650 (360 [1,200])	600 (350 [1,030])	588 (327.5 [1,025])	920 (560 [1,580])	.32
Family income at age 15 years (R$)^b^	836 (480 [1,500])	960 (395 [1,250])	872 (680 [1,800])	1480 (901 [2,183])	.10

p = interquartile range.

a We calculated *p* values using the Kruskal-Wallis test, because the variables were asymmetric.

b One R$ is equivalent to US $0.5.

**Table 2:** Anthropometric characteristics of the sample (N = 80)

	Girls		Boys		*p* value^a^
	Eutrophic	Obese	Eutrophic	Obese	
	(mean ± SD)	(mean ± SD)	(mean ± SD)	(mean ± SD)	
BMI (kg/m^2^)^a^	17.7 ± 2.2	27.8 ± 3.2	17.1 ± 1.6	25.5 ± 3.2	<.001
BMI (kg/m^2^)^b^	20.6 ± 2.2	31.9 ± 3.9	19.4 ± 1.6	30.5 ± 4.6	<.001
Weight (kg)^b^	52.6 ± 6.9	85.2 ± 13.3	54.4 ± 8.8	87.9 ± 17.2	<.001
Height (cm)^b^	159.6 ± 5.5	163.2 ± 5.9	167.1 ± 10.6	169.3 ± 9.2	.002
Subscapular skinfold (mm)^b^	12.3 ± 4.7	30.0 ± 7.5	7.6 ± 2.0	25.8 ± 7.5	<.001
Triceps skinfold (mm)^b^	16.9 ± 6.0	32.8 ± 5.7	9.2 ± 4.7	27.4 ± 8.6	<.001

BMI = body mass index; SD = standard deviation.

a Information collected at age 11 years

b Information collected at age 15 years

**Table 3:** Reasons for obesity etiology reported by adolescent and their mothers (N = 80)

BMI (11 and 15 years)	Family income	
	High-income adolescents	Low-income adolescents
Obese	Genetic factors	Gene family/identity
	Life events	Sociocultural pressure
	Individual/moral characteristics^a^	Life events
Eutrophic	Individual/moral characteristics	Gene family Individual/moral characteristics

a Individual/moral characteristics = lack of willpower; overeating, sloth, sedentary lifestyles, fat diet.
